# The transcriptional activity of Gli1 is negatively regulated by AMPK through Hedgehog partial agonism in hepatocellular carcinoma

**DOI:** 10.3892/ijmm.2014.1847

**Published:** 2014-07-10

**Authors:** QIURAN XU, XIN LIU, XIN ZHENG, YINGMIN YAO, MAODE WANG, QINGGUANG LIU

**Affiliations:** 1 Department of Hepatobiliary Surgery, The First Affiliated Hospital of Medical College of Xi’an Jiaotong University, Xi’an, Shaanxi 710061, P.R. China; 2 Department of Neurosurgery, The First Affiliated Hospital of Medical College of Xi’an Jiaotong University, Xi’an, Shaanxi 710061, P.R. China

**Keywords:** 5′ adenosine monophosphate-activated protein kinase, glioma-associated oncogene 1, hepatocellular carcinoma, Hedgehog, interaction

## Abstract

The aberrant activation of the Hedgehog (Hh) signaling pathway has been implicated in a variety of malignancies, including hepatocellular carcinoma (HCC). The mammalian 5′ adenosine monophosphate-activated protein kinase (AMPK) plays a crucial role in cellular energy homeostasis. However, the interaction between the Hh and AMPK signaling pathways has not been investigated to date. In the present study, to the best of our knowlege, we report for the first time the negative regulation of glioma-associated oncogene 1 (Gli1), an important downstream effector of Hh, by the AMPK signal transduction pathway. Immunoprecipitation and GST-pull down assay showed a direct interaction between AMPK and Gli1. The overexpression of AMPK induced the downregulation of Gli1 expression, while the knockdown of AMPK upregulated Gli1 expression in a relatively short period of time (24 h or less). Our data suggest that AMPK may function as an upstream molecule that regulates Gli1 expression. Therefore, AMPK may play a role in the Hh signaling pathway, through which it regulates tumorigenesis.

## Introduction

Hepatocellular carcinoma (HCC) is the most common form of liver cancer and the third leading cause of cancer-related mortality worldwide (1). The incidence of HCC has increased in recent years. The Hedgehog (Hh) signaling pathway plays an important role in embryonic development, as well as in the regulation of a variety of cellular functions. Aberrant Hh signaling is associated with a variety of cancers, including HCC. Glioma-associated oncogene 1 (Gli1) is an important downstream effector of Hh. It acts as a transcription factor, participating in the promotion of cell growth and the inhibition of apoptosis. The transcription of components of the Hh signaling pathway and related molecules has been reported to be increased in some cases of HCC (2). Suh *et al* (3) demonstrated that the Hh signaling pathway plays a conserved role in inhibiting fat formation. Teperino *et al* (4) also demonstrated that the Hh signaling pathway stimulates metabolic reprogramming towards a Warburg-like glycolytic state, and specifically blocks the adipogenesis of white adipocytes, but not brown adipocytes. This effect, mediated by a rapid non-canonical Smo-Ca^2+^-Ampk signaling arm, causes robust glucose uptake in mouse and human myocytes, and is induced by several canonical Hh signaling inhibitors.

5′ Adenosine monophosphate (AMP)-activated protein kinase(AMPK) plays a crucial role in cellular energy homeostasis, and is responsive to stimulation by nutrients, stress or exercise (5–7). The disruption of this balance is associated with a number of diseases, such as diabetes and cancer (8,9). AMPK is a heterotrimeric serine/threonine protein kinase composed of 3 subunits: a catalytic subunit (α), a scaffolding subunit (β) and an AMP-sensing subunit (γ). Its kinase activity is controlled by the AMP/ATP ratio and some upstream kinases, such as liver kinase B1 (LKB1), TGF-β-activated kinase 1 (TAK1) and Ca^2+^/calmodulin-dependent protein kinase kinase (CaMKK) (10–15). AMPK controls cell metabolism and growth in response to changes in nutrient availability by phosphorylating a variety of substrates in cells, including acetyl-CoA carboxylase (ACC), forkhead box O3 (FOXO3) and tuberous sclerosis complex 2 (TSC2) (16–18). AMPK also regulates gene transcription through direct association with chromatin and the phosphorylation of histone H2B at serine 36 (19). However, it remains unclear as to whether an interaction exists between the AMPK and Hh pathways.

Since both the AMPK pathway and Hh signaling pathway affect cellular metabolism, we hypothesized that these two pathways may interact with each other. The results of the present study demonstrate that AMPK expression negatively correlates with the expression of both Sonic hedgehog (Shh) and Gli1 in HCC tissues. The treatment of HepG2 cells with smoothened agonist (SAG) or cyclopamine (a specific inhibitor of Hh signaling) resulted in a negative correlation between AMPK and Gli1 expression, which was observed in a relatively short period of time (24 h or less). Furthermore, the overexpression of AMPK induced the downregulation of Gli1 expression, while the knockdown of AMPK upregulated Gli1 expression in a relatively short period of time (24 h or less).

Thus, AMPK may play an important role in the Hh signaling pathway. Understanding the relationship between AMPK and Hh signaling is important in order to elucidate the mechanisms through which they regulate HCC pathogenesis.

## Materials and methods

### Cell lines, plasmids, tissue samples, chemicals and culture media

Cell lines and culture conditions were as follows: HepG2 (from ATCC, Manassas, VA, USA) were cultured in Dulbecco’s modified Eagle’s medium (DMEM; HyClone, Logan, UT, USA) supplemented with 10% (v/v) fetal bovine serum (FBS; Gibco Life Technologies, Carlsbad, CA, USA); and 293T cells (from the National Platform of Experimental cell Resources for Sci-Tech, Beijing, China) were cultured in RPMI-1640 medium (HyClone) supplemented with 10% (v/v) FBS. AMPKα1 cDNA fragments were PCR-amplified and cloned into the pS-Flag-SBP (SBP) vector. The human Gli1 expression vector, pcDNA3-Gli1, and the pIRES2-S-SBP-FLAG plasmid were kindly provided by Dr Xin Zheng (Deparment of Department of Hepatobiliary Surgery, the First Affiliated Hospital of Medical College of Xi’an Jiaotong University, Xi’an, China). Vector PLKO was purchased from Addgene; it is a replication-incompetent lentiviral vector selected by the TRC (The RNAi Consortium) for the expression of shRNAs. GFP-AMPK plasmid was also kindly provided by Dr Xin Zheng, which had a GFP tag.

A total of 63 patients with HCC were enrolled in this study between January 2009 and October 2009, including 49 males and 14 females who had not received pre-operative chemotherapy or embolization. Following routine X-ray, abdominal ultrasonography and computed tomography, all patients underwent liver resection, including curative resection for early HCC and palliative resection for advanced HCC. Tumor tissue and matched adjacent normal tissue specimens (>2 cm distance to the resection margin) were collected and immediately stored in liquid nitrogen for RT-PCR and paraformaldehyde for immunohistochemistry. Clinical data were obtained from the medical records. The histopathological Edmonson classification, clinical tumor-node-metastasis (TNM) grading, maximum tumor diameter and the adjacent normal tissues were all confirmed by an experienced pathologist who was blinded to the clinical information The following clinicopathological parameters of the patients were investigated: age, hepatitis B virus surface antigen HBsAg (+), cirrhosis (+), serum alpha fetoprotein (AFP) levels (>400 ng/ml), tumor size (>5 cm), primary tumor (II and III), clinical tumor-node-metastasis (TNM) grading (III and IV), differentiation, portal vein tumor thrombosis (PVTT) (+), lymph node invasion (+), encapsulation (+)”. Univariate and multivariate analyses of the clinicopathological characteristics were carried out using the Cox hazard model.

Written informed consent was obtained from all patients enrolled in this study. The Xi’an Jiaotong University Ethics Committee approved all protocols according to the Helsinki Declaration of 1975.

### Chemical reagents and antibodies

Anti-AMPKα1 and anti-actin antibodies were purchased from Cell Signaling Technology (Danvers, MA, USA). Anti-Gli1, anti-Shh and anti-HA antibodies were purchased from Santa Cruz Biotechnology (Santa Cruz, CA, USA).

### Immunohistochemical staining

Immunohistochemistry was performed on paraformaldehyde-fixed paraffin sections. The sections were dewaxed and dehydrated. Following rehydration, endogenous peroxidase activity was blocked for 30 min using a methanol solution containing 0.3% hydrogen peroxide. Following antigen retrieval in citrate buffer, the sections were blocked overnight at 4°C, then separately incubated with the primary antibodies directed against Gli1 and AMPK at 4°C overnight. The primary antibody was detected using biotinylated secondary antibodies (Zhongshan Golden Bridge Biotechnology Ltd., Co., Beijing, China) according to the manufacturer’s recommendations. The staining of the sections was performed using the avidin-biotin-peroxidase complex for Gli1, Shh and AMPK (SABC method). The sections were visualized using diaminobenzidine and counterstained with hematoxylin, then dehydrated in alcohol and xylene and mounted onto glass slides.

All sections were assessed independently by two experienced pathologists. The staining results for the all proteins (Gli, Shh and AMPK) were semi-quantitatively expressed by an immunohistochemical score combined with the percentage of tumor cells showing specific immunoreactivity. The staining intensity was expressed in 4 grades: 0, none; 1, weak; 2, oderate; and 3, strong. The percentage of positive carcinoma cells was expressed in the following grades: 0, <5%; 1, 6–25%; 2, 26–50%; 3, 51–75%; and 4, >75%. The staining intensity and average percentage of positive tumor cells were assayed for 10 independent high magnification (×400) fields. The total score was calculated by multiplying the staining intensity and the percentage of positive tumor cells. Sections with a total score of >1 were defined as exhibiting positive staining for the above-mentioned proteins.

### Cell lysis, immunoprecipitation and western blot analysis

Cell transfection, protein extract preparation, immunoprecipitation and western blot analysis were performed as previously described in the study by Kim *et al* (20,21). Briefly, for immunoprecipitation, the cells were lysed with ice-cold NETN buffer (20 mM Tris-HCl, pH 8.0, 100 mM NaCl, 1 mM EDTA and 0.5% Nonidet P-40) containing 10 mM NaF and 50 mM b-glycerophosphate, and then subjected to sonication for 12 sec. The supernatants were incubated with he indicated antibodies and protein G-conjugated Sepharose beads (Amersham Pharmacia Biotech Inc., Piscataway, NJ, USA). The precipitates were washed 3 times with NETN, and subjected to SDS-PAGE and western blot analysis with the indicated antibodies. To examine the AMPK levels, the cell pellets were lysed with 400 μl NETN100 buffer. Following centrifugation, the supernatants were named as 100 mM NaCl samples. The insoluble pellets were collected, washed with ice-cold PBS, and incubated with 400 μl NETN300 buffer on ice. Following centrifugation, the supernatants were named as 300 mM NaCl samples. The remaining pellets were washed twice with ice-cold PBS and then treated with 200 μl 0.2N HCl. The supernatants were neutralized with 40 μl 1N NaOH, and named as 0.2N HCl fractions. Each fraction sample was loaded onto 7.5% SDS-PAGE gels for western blot analysis with the indicated antibodies.

### RNA extraction and RT-qPCR

The expression of Gli1 was determined by the reverse transcription of total RNA, followed by quantitative PCR analysis. Total RNA (1 μg) was reverse transcribed with random hexamers using SuperScript II Reverse Transcriptase (Invitrogen, Carlsbad, CA, USA) according to the manufacturer’s instructions. Real-time PCR was performed on a Bio-Rad iCycler using iQ SYBR-Green (Bio-Rad Laboratories, Hercules, CA, USA) with the following primers: Gli1 forward, 5′-GAAGGTGAAGGTCGGAGT-3′ and reverse, 5′-GTCCAGGCTGGCATCCGACA-3′; and GAPDH forward, 5′-GAAGGTGAAGGTCGGAGT-3′ and reverse, 5′-GAAGA TGGTGATGGGATTTC-3′.

### DNA constructs and mutagenesis

PCR-amplified human AMPKα1 was cloned into the pS-Flag-SBP (SBP) vector. AMPKα1 shRNA #1-resistant and shRNA #2-resistant were created using the QuikChange Site-Directed Mutagenesis kit (Stratagene, La Jolla, CA, USA).

AMPKα1 shRNA #1 was generated with GATGGAATAT GTCTCAGGAGG. AMPKα1 shRNA #2 was generated with ATGATGTCAGATGGTGAATTT. We used the following primers: AMPKα1 shRNA #1-resistant forward, 5′-GATAT TTTCATGGTaATGGAgTAcGTgagtGGtGGAGAGCTATT TGA and reverse, 5′-TCAAATAGCTCTCCaCCactcACg TAcTCCATtACCATGAAAATATC; and shRNA #2-resistant forward, 5′-GGTCTTTCAAACATGATGagaGAaGGaGAgT TcTTAAGAACAAGTTG and reverse, 5′-CAACTTGTTCTT AAgAAcTCtCCtTCtctCATCATGTTTGAAAGACC.

### GST-pull down assay

For the GST pull-down assay, 1 μg of GST-AMPK or GST as a control was incubated with the cell lysates from the 293T cells overexpressing HA-tagged Gli1. Glutathione beads were then added and followed by incubation for 2 h. The bound proteins were eluted with sample loading buffer and analyzed by immunoblotting with HA antibodies. For endogenous immunoprecipitation, the 293T cell lysates were immunoprecipitated with normal mouse IgG as a control, followed by incubation with protein A beads. The bound proteins were subjected to immunoblot analysis with AMPK antibody.

### Transfection

Tgh 293T cells were grown in DMEM containing 10% FBS. The Cells (2×10^6^) were seeded in 10-cm dishes 24 h prior to transfection and transfected with 5 μg of the AMPK plasmid using the Lipofectamine 2000 reagent (Invitrogen) according to manufacturer’s instructions.

### Statistical analysis

Throughout the study, the distribution of data points is expressed as the mean ± standard error of the mean. The Mann-Whitney U test was used for statistical analysis unless otherwise indicated. The relationship between the clinicopathological characteristics of the patients and Gli1 protein expression was analyzed using the Cox hazard model. Additionally, the Spearman rank test was also used to analyze the correlation between AMPK protein expression and the expression of Shh and Gli1. Values of P<0.05 were considered to indicate statistically significant differences.

## Results

### Expression of Gli1, Shh and AMPK in HCC

**Figure 1:**
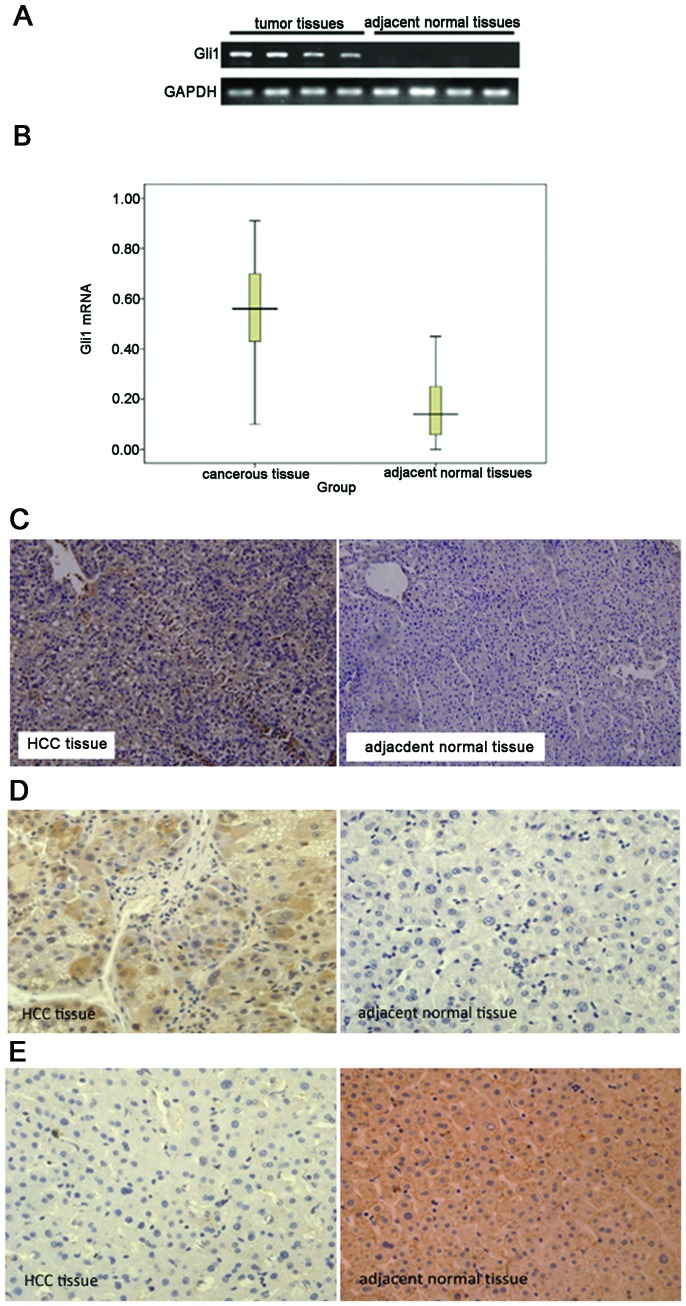
(A) Glioma-associated oncogene 1 (Gli1) mRNA levels were measured by RT-qPCR in tumor and adjacent normal tissues. Expression levels were normalized to the GAPDH level in each sample. (B) mRNA expression of Gli1 in HCC tissues and adjacent normal tissues. (C) Protein expression pattern of Gli1 in HCC tumor tissues and adjacent normal tissues by immunohistochemistry. (D) Shh protein expression in HCC tissues and adjacent normal tissues. (E) Expression pattern of 5′ adenosine monophosphate-activated protein kinase (AMPK) protein in HCC tissues and adjacent normal tissues by immunohistochemistry. HCC, hepatocellular carcinoma.

To evaluate Gli1 expression in HCC, a total of 63 pairs of HCC and adjacent tissues from HCC patients were examined by RT-qPCR and immunohistochemistry. Gli1 mRNA was detected in 52 HCC samples, but only in 16 of the adjacent normal samples (Fig. 1A). The expression of Gli1 mRNA in the HCC tissues was significantly higher than that in the adjacent normal tissues (Fig. 1B). Gli1 protein levels in the HCC and adjacent normal tissues were measured using a Gli1-specific antibody. Gli1 protein was detected in 57 of the 63 tumor tissues (90.47%), including 17 (26.98%) highly positive (+++) cases. By contrast, Gli1 protein expression was only observed in 21 of the 63 normal tissues (33.33%), and none of these normal tissues was scored as highly positive (Fig. 1C, Table I).

The protein expression of Shh was examined by immunohistochemistry. As shown in Fig. 1D, Shh was mainly expressed in the cytoplasm. Quantitative analysis indicated that the level of Shh in the HCC tissues was significantly higher than that in the adjacent normal tissues (P<0.001). In addition, a negative correlation between AMPK and Shh expression was confirmed in the HCC tissues using the Spearman rank test (r=−0.574, P<0.05).

Similarly, the expression of AMPK protein was determined by immunohistochemistry. As shown in Fig. 1E, AMPK was mainly located on the cytomembrane. Quantitative analysis revealed that the level of AMPK in the HCC tissues was significantly lower than that in the adjacent normal tissues (4.40±3.68 vs. 0.47±1.01; P<0.05).

Furthermore, the Spearman rank test revealed a negative correlation between Gli1 and AMPK expression levels (r=−0.429, P<0.05).

### Gli1 expression correlates with the prognosis of HCC patients

**Figure 2:**
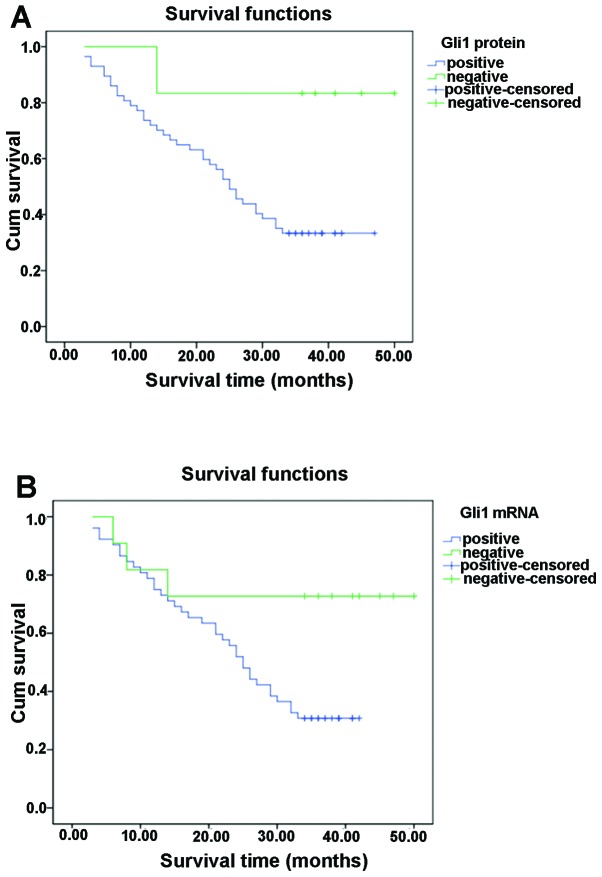
Glioma-associated oncogene 1 (Gli1) expression level affects the prognosis of patients with hepatocellular carcinoma (HCC). (A) Relationship between Gli1 protein levels and overall survival in HCC patients. (B) Relationship between Gli1 mRNA levels and overall survival in HCC patients.

We also analyzed the correlation between Gli1 expression and patient survival, which was carried out by univariate and multivariate analyses adjusted for clinicopathological parameters. Cox regression analysis revealed that there was a significant correlation between Gli1 expression and tumor invasiveness, including histological differentiation, portal vein tumorous thrombogenesis, lymph node invasion and TNM stage (Table II). Using Kaplan-Meier analysis, we found that the patients with positive Gli1 expression had poor overall survival prognosis (Fig. 2, Table III). These results suggest that Gli1 is a useful prognostic marker for HCC patients.

### Correlation between AMPK and Gli1 is reversed by treatment with cyclopamine or SAG

**Figure 3:**
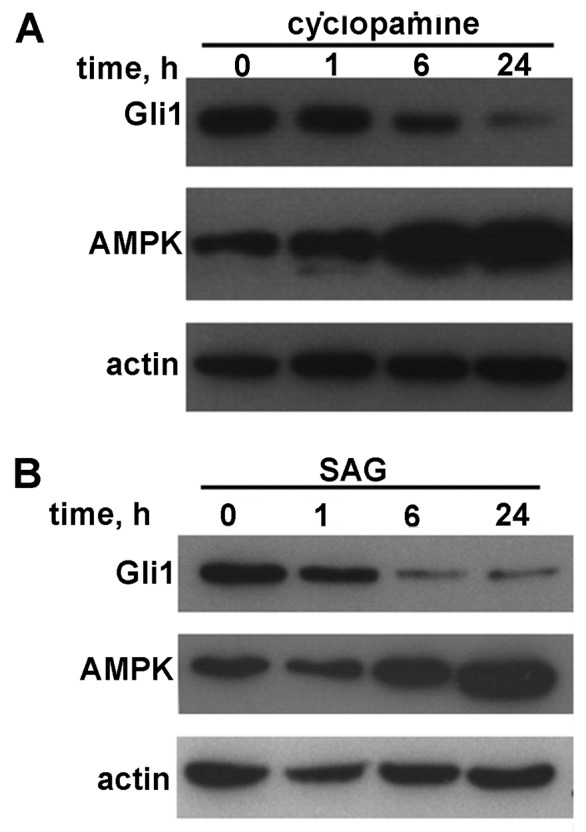
HepG2 cells was treated with (A) cyclopamine (100 nM) or (B) smoothened agonist (SAG; 200 nM) and collected at 0, 1, 6 and 24 h. Cell lysates were subjected to western blot analysis.

The HepG2 cells were treated with cyclopamine (a specific inhibitor of Hh signaling) or SAG. The cell lysates were then immunoblotted with Gli1 and AMPK antibodies. As shown in Fig. 3, a negative correlation between AMPK and Gli1 was observed in a relatively short period of time (24 h or less).

### Gli1 directly interacts with AMPK

**Figure 4:**
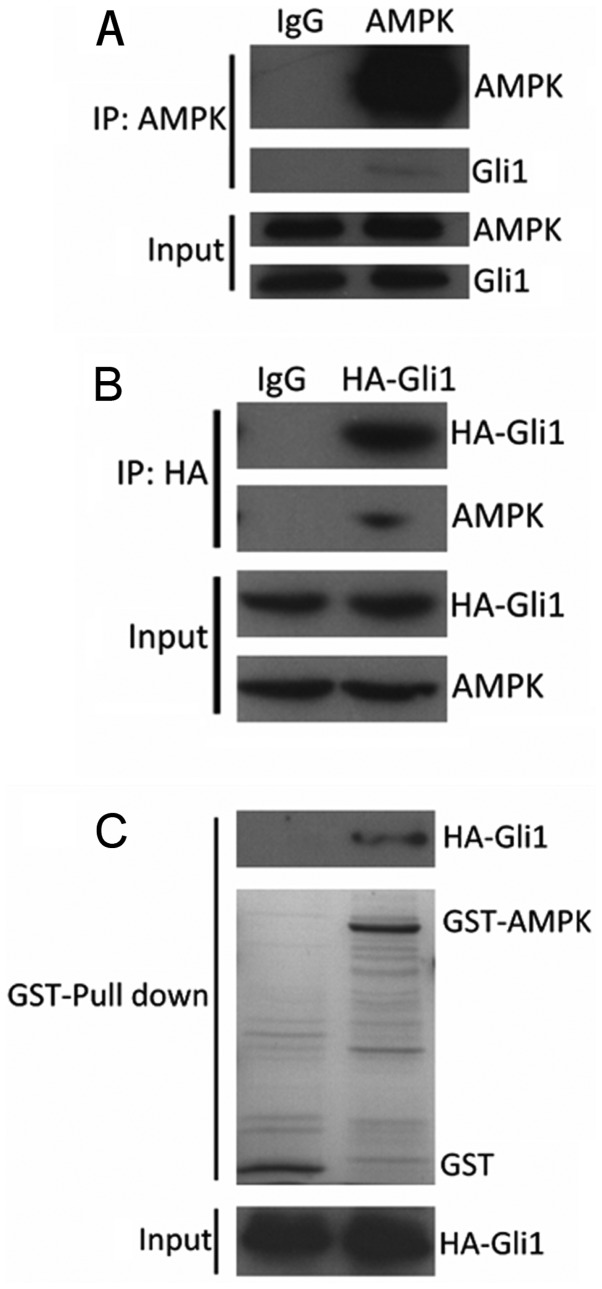
Glioma-associated oncogene 1 (Gli1) directly interacts with 5′ adenosine monophosphate-activated protein kinase (AMPK). (A) 293T cell lysates were immunoprecipitated with AMPK antibody or normal mouse IgG and then immunoblotted with AMPK and Gli1 antibodies. (B) 293T cells were transiently transfected with HA-Gli1, and cell lysates were immunoprecipitated with anti-HA resin, then immunoblotted with HA and AMPK antibodies. (C) 293T cells were transfected with HA-Gli1. The cell lysates were subjected to a pull-down assay with GST or GST-AMPK.

To further understand the functional relationship between AMPK and Gli1, we examined the physical interaction between these two molecules using immunoprecipitation. As shown in Fig. 4A, Gli1 protein was precipitated from the 293T cells lysates using AMPK-specific antibody. We also examined whether AMPK can be precipitated by Gli1 antibody. As Gli1-specific antibody for immunoprecipitation is not available, we transfected the 293T cells with HA-Gli1. The cell lysates were subjected to incubation with HA antibody and immunoblotted with AMPK antibody. We found that HA-Gli1 interacted with AMPK (Fig. 4B). To examine the direct interaction between AMPK and Gli1, GST-AMPK and HA-Gli1 were purified for the GST pull-down assay. As shown in Fig. 4C, GST-AMPK interacted with HA-Gli1 in the 293T cells. These results indicated that AMPK interacted with Gli1 *in vitro*.

### AMPK regulates Gli1 expression

**Figure 5:**
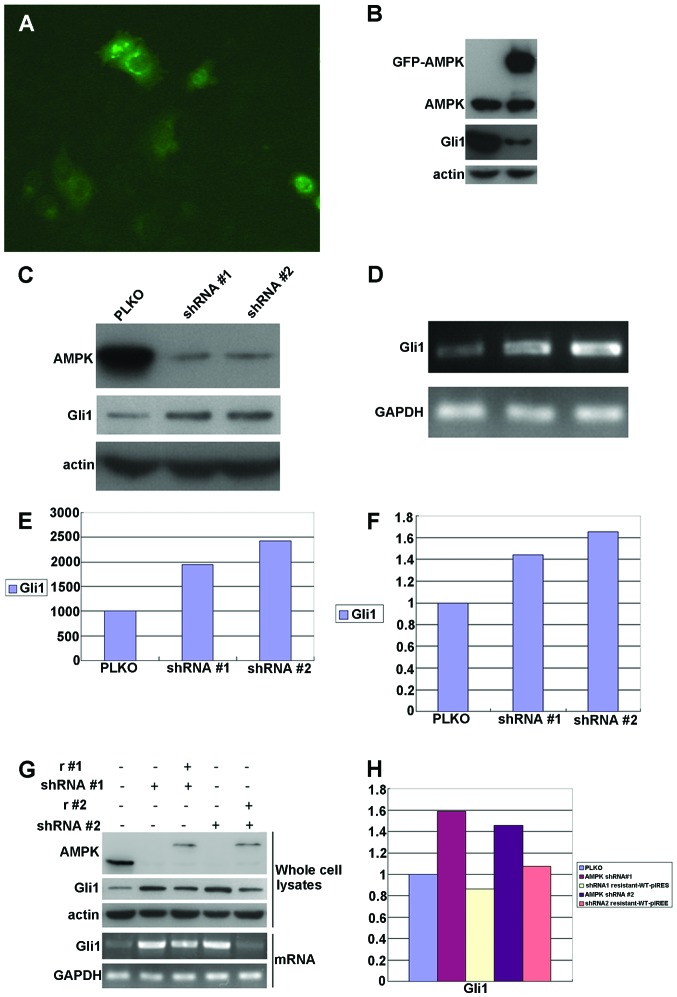
5′ Adenosine monophosphate-activated protein kinase (AMPK) regulates glioma-associated oncogene 1 (Gli1) expression. (A) HepG2 cells were transiently transfected with GFP-AMPK. (B) Overexpression of AMPK in HepG2 cells decreased Gli1 expression. (C) Konckdown of AMPK using shRNA in HepG2 cells induced an increase in Gli1 expression. (D, E and F) HepG2 cells were transduced with AMPK shRNA #1 or shRNA #2 or PLKO (a replication-incompetent lentiviral vector for the expression of shRNAs used as a control). Twenty-four hours after infection, RT-PCR and qPCR were performed. (G and H) Gli1 expression was analyzed by western blot analysis using whole cell lysates (top) and RT-PCR, qPCR using total RNA (bottom) from HepG2 cells. HepG2 cells with or without depleted AMPK and reconstituted expression of AMPK showed differential expression levels of Gli1.

To investigate whether AMPK regulates Gli1 expression, AMPK was overexpressed in the HepG2 cells by transfecting the cells with the pIRES-S-SBP-FLAG-AMPK vector. Gli1 expression was examined in the transfected cells (Fig. 5A). It was found that Gli1 expression was significantly downregulated in the AMPK-overexpressing cells (Fig. 5B). We also examined the effects of AMPK downregulation by shRNA knockdown. The HepG2 cells were transfected with recombinant lentiviruses expressing either AMPK shRNA #1 or shRNA #2 or vector PLKO as a control. Both ‘targeted’ shRNAs decreased AMPK expression in the HepG2 cells (Fig. 5C; >80% knockdown). AMPK knockdown significantly increased the levels of Gli1 mRNA and protein (Fig. 5C–F).

To further verify the specificity of AMPK shRNAs and the regulatory role of AMPK in Gli1 expression, a mutated version of AMPK was expressed in the HepG2 cells. This AMPK mutant is resistant to AMPK shRNAs, but can express functional AMPK in HepG2 cells. We found that Gil1 expression in the HepG2 cells was completely rescued by the expression of AMPK mutant. The effects were observed at both the mRNA and protein level (Fig. 5G and H). These results confirmed that Gli1 was specifically regulated by AMPK.

## Discussion

The Hh signaling pathway plays an important role in embryonic development, as well as in the regulation of a variety of cellular functions (22–25). Aberrant Hh signaling is associated with a variety of cancers, including glioma (26,27), glioblastoma (28,29), breast cancer (30,31) and pancreatic adenocarcinoma (32,33) among others (34–36).

In mammals, the Hh signaling pathway mainly consists of Hh ligands [Desert hedgehog (Dhh), Indian hedgehog (Ihh) and Shh], two transmembrane proteins [patched 1 (Ptch) and smoothened, frizzled class receptor (Smo)], the nuclear transcription factor, Gli (Gli1, Gli2 and Gli3), and downstream target genes. When this pathway is activated, ligand binding to the inhibitory cell surface receptor, Ptch, initiates Smo translocation to the tip of the cilium and liberates the zinc finger family transcription factors, Gli1, Gli2 and Gli3, from proteasomal degradation and inhibitory tethering, leading to the activation of Gli target genes (37–41), which exert further effects on cell growth, proliferation and differentiation.

In the present study, using immunohistochemical analysis, we first confirmed that the Shh and Gli1 proteins were expressed at significantly higher levels in the HCC tissues than in the adjacent normal tissues. This result suggests that Hh signaling, which plays a role in liver embryonic development and differentiation, was aberrantly activated and that this may lead to tumorgenesis in HCC.

In addition, we analyzed the correlation between Gli1 expression and tumor pathological characteristics and patient prognosis. The results revealed that Gli1 expression in the highly aggressive (including histological differentiation, portal vein tumorous thrombogenesis, lymph node invasion and TNM stage) HCC group was significantly higher than that in the less aggressive HCC group. Furthermore, there was a significant negative correlation between Gli1 expression and patient prognosis. These results suggest that Hh signaling plays a critical role in hepatocarcinogenesis. However, the exact molecular mechanisms of Hh signaling in HCC progression remain unclear.

It is known that cancer cells show enhanced glycolysis and inhibition of oxidative phosphorylation, even in the presence of sufficient oxygen (aerobic glycolysis) (42). AMPK is a key regulator of cellular metabolism and is involved in the pathogenesis of several diseases (8,9). However, to the best of our knowlwedge, there is no report to date on the possible interactions between AMPK and Hh signaling.

Recent studies have indicated that Hh signaling rewires cellular metabolism. There is a cilium-dependent Smo-Ca^2+^-Ampk axis that triggers rapid Warburg-like metabolic reprogramming within minutes of activation, which is required for proper metabolic selectivity and flexibility. In a previous study, Teperino *et al* (4) demonstrated that Smo modulators can uncouple the Smo-Ampk axis from the canonical signaling pathway, and identified cyclopamine as one of the new class of ‘selective partial agonists’, capable of the concomitant inhibition of canonical Hh signaling and the activation of non-canonical Hh signaling.

The present study revealed a negative correlation between AMPK and Gli1 expression. Of note, when the HepG2 cells were treated with cyclopamine or SAG, the Gli1 expression level decreased, but the AMPK expression level increased in a relatively short period of time (24 h or less). Furthermore, the overexpression of AMPK downregulated Gli1 expression, while the knockdown of AMPK upregulated Gli1 expression in a relatively short period of time (24 h or less). On the basis of these findings, we suggest that AMPK may play a role in the Hh signaling pathway by interacting with Shh and Gli1, and this interaction may affect cellular metabolism and tumorgenesis. Further studies are required to elucidate the molecular mechanisms involved in this process.

## Figures and Tables

**Table I:** Expression of Gli1 protein in the HCC tumor tissues and adjacent normal tissues.

		Gli1 expression level						
					
Pathological type	n	−	+	+ +	+ + +	Positive n (%)	χ^2^	P-value
HCC tissues	63	6	8	32	17	57 (90.47)		
Adjacent normal tissues	63	42	18	3	0	21 (33.33)	43.6	<0.05

Gli1, glioma-associated oncogene 1; HCC, hepatocellular carcinoma.

**Table II:** Prognostic factors in the Cox proportional hazards model.

Parameters	RR	95% CI	Wald	P-value
Gender	0.819	0.127–5.274	0.044	0.834
Age	1.033	0.205–5.196	0.002	0.968
HBsAg	0.310	0.057–1.675	1.851	0.174
Cirrhosis	0.237	0.033–1.721	2.027	0.155
Serum AFP (>400 ng/ml)	0.530	0.062–4.494	0.339	0.560
Tumor size (>5 cm)	0.728	0.181–2.923	0.200	0.655
Differentiation	15.197	2.039–113.291	7.048	0.008^a^
PVTT	6.041	1.395–26.162	5.784	0.016^a^
Lymph node invasion	0.032	0.003–0.369	7.627	0.006^a^
Encapsulation	2.484	0.435–14.180	1.048	0.306
Primary tumor (II and III)	3.105	0.395–24.435	1.159	0.282
TNM stage (III and IV)	75.634	2.757–2075E3	6.554	0.010^a^
Gli1 mRNA	22.298	2.110–235.510	6.663	0.010^a^

a P<0.05, indicating statistical significance.

RR, relative risk; Wald, Wald value; HBsAg, hepatitis B virus surface antigen; AFP, alpha fetoprotein; PVTT, portal vein tumor thrombosis; Gli1, glioma-associated oncogene 1.

**Table III:** Univariate prognostic factors in Kaplan-Meier survival analysis.

Characteristics	N	Deaths	Median/M	SE	Log-rank	P-value
Gender						
Male	49	31	26.00	3.50		
Female	14	8	25.58	3.74	0.124	0.725
Age (years)						
≤45	15	10	24.00	7.24		
>45	48	29	26.00	2.47	0.201	0.654
HBsAg						
Positive	50	33	25.00	2.80		
Negative	13	6	29.00	3.73	0.933	0.334
Cirrhosis						
Positive	51	36	24.00	2.40		
Negative	11	3	30.00	2.83	5.466	0.019^a^
Serum AFP/(ng/ml)						
≤400	16	7	31.00	4.27		
>400	47	32	25.00	2.06	2.500	0.114
Tumor size (cm)						
≤5	21	13	28.00	6.29		
>5	42	26	26.00	2.69	0.004	0.953
Differentiation						
Well and moderate (I + II)	49	25	33.00	2.46		
Poor and none (III + IV)	14	14	12.00	2.49	18.669	0.000^a^
PVTT						
Positive	21	18	21.00	5.34		
Negative	42	21	29.00	2.63	4.924	0.026^a^
Lymph node invasion						
Positive	27	22	19.00	4.33		
Negative	36	17	26.00	2.84	6.870	0.009^a^
Encapsulation						
Positive	40	29	24.00	2.37		
Negative	23	10	28.00	3.11	3.645	0.056
Primary tumor						
T1+T2	19	6	40.00	3.51		
T3+T4	44	33	23.00	3.27	10.548	0.001^a^
TNM stage						
I+II	19	2	45.00	2.75		
III+IV	44	37	21.00	3.32	23.612	0.000^a^
Gli1 protein						
Positive	57	38	24.00	2.15		
Negative	6	1	41.00	3.19	3.996	0.046^a^
Gli1 mRNA						
Positive	52	36	25.00	1.80		
Negative	11	3	38.00	2.81	4.230	0.040^a^

a P<0.05, indicating statistical significance.

HBsAg, hepatitis B virus surface antigen; AFP, alpha fetoprotein; PVTT, portal vein tumor thrombosis; Gli1, glioma-associated oncogene 1.
